# Integrative Gene Selection on Gene Expression Data: Providing Biological Context to Traditional Approaches

**DOI:** 10.1515/jib-2018-0064

**Published:** 2018-12-22

**Authors:** Cindy Perscheid, Bastien Grasnick, Matthias Uflacker

**Affiliations:** Hasso Plattner Institute, Digital Engineering Faculty, University of Potsdam, Potsdam, Germany

**Keywords:** Gene Expression Data Analysis, Integrative Gene Selection, Pattern Recognition, Prior Knowledge, Knowledge Bases

## Abstract

The advance of high-throughput RNA-Sequencing techniques enables researchers to analyze the complete gene activity in particular cells. From the insights of such analyses, researchers can identify disease-specific expression profiles, thus understand complex diseases like cancer, and eventually develop effective measures for diagnosis and treatment. The high dimensionality of gene expression data poses challenges to its computational analysis, which is addressed with measures of gene selection. Traditional gene selection approaches base their findings on statistical analyses of the actual expression levels, which implies several drawbacks when it comes to accurately identifying the underlying biological processes. In turn, integrative approaches include curated information on biological processes from external knowledge bases during gene selection, which promises to lead to better interpretability and improved predictive performance. Our work compares the performance of traditional and integrative gene selection approaches. Moreover, we propose a straightforward approach to integrate external knowledge with traditional gene selection approaches. We introduce a framework enabling the automatic external knowledge integration, gene selection, and evaluation. Evaluation results prove our framework to be a useful tool for evaluation and show that integration of external knowledge improves overall analysis results.

## 1 Introduction

Recent advances in high-throughput sequencing technologies allow a more in-depth analysis of the human genome. RNA-Sequencing, in particular, measures the complete gene activity, i.e. gene expression levels, from specific tissues [1]. Researchers use this data to investigate on the molecular characteristics of diseases, e.g. cancer. Analyzing interactions of genes and their associations to diseases leads to their deeper understanding, helps to improve diagnosis and results in more effective treatment [2].

The high dimensionality of gene expression data containing tens of thousands of genes as features coupled with a low number of samples, noise and redundancy poses challenges to its processing [3]. A feasible computational data analysis requires measures for dimensionality reduction, which is addressed by feature selection – in this context referred to as gene selection. Gene selection approaches rank genes according to a specific measure, and an optimal gene ranking reflects the biological processes inherent in the data. It is a hard task to identify these processes based on data statistics alone. For example, two genes can show very similar expression patterns in the dataset, but actually correlate just by chance and participate in different biological processes. Traditional approaches for gene selection would see a correlation of these two genes as they rely on observations made in the data alone and miss the biological context.

There are two options for gene selection to avoid undesired behavior like this: On the one hand, researchers apply more complex gene selection, e.g. based on machine learning, that is more likely to identify the underlying biological processes. However, more complex algorithms lead to more computational runtime and decrease researchers’ understanding of what is going on during computation. When applying traditional gene selection, researchers now face the challenge of finding an approach that a) balances a high result set accuracy with feasible computational runtime, and b) remains transparent in the way it generates its findings. On the other hand, literature argues that gene selection can profit from including the biological context early into its analyses [4]. At the same time, publicly available knowledge bases provide machine-readable, curated repositories and meta-repositories of biological knowledge in the form of gene-disease-associations, pathway information, and ontologies [5], [6], [7]. We argue that this available information can be used to provide gene selection approaches with the biological context they need. Gene selection can profit from integrating external knowledge on biological processes, as this will lead to the delivery of biologically meaningful results and a reduced computational complexity that facilitates a transparent processing and feasible execution runtimes. Consequently, the aim of this paper is to investigate on how the integration of external knowledge can improve the analysis of gene expression data, e.g. in terms of biological interpretability, performance in classification, and runtime durations compared to traditional approaches.

Our contribution is two-fold: In order to examine the impact of external knowledge bases on the analysis, we present an own straightforward approach to flexibly combine external knowledge and traditional approaches as desired. We conduct a study that measures the performance of cancer type classification on multiple datasets from The Cancer Genome Atlas (TCGA), comparing traditional approaches and combinations thereof with external knowledge according to our own approach. In addition, we introduce a framework that allows the flexible combination and comparison of knowledge bases with traditional approaches, integrating multiple knowledge bases, a wide range of the existing traditional approaches, classifiers and evaluation metrics.

The remainder of the paper is structured as follows: Section 2 presents related work on both traditional and integrative gene selection. Section 3 presents our framework in detail, including the distinct processing steps and our own approaches to integrate external knowledge into gene selection. Section 4 describes the experiments conducted and provides evaluation results. Section 5 discusses these results in a broader context. Section 6 summarizes our main findings.

## 2 Related Work

Combining traditional gene selection with external knowledge is an emerging field of research. Currently, however, traditional approaches are predominantly used because existing integrative approaches are mostly manually built and specific to a certain use case. In the following, we first highlight the current state of the art for traditional approaches, and afterwards concentrate on existing integrative approaches.

### 2.1 Traditional Gene Selection

Literature classifies Gene selection approaches according to their characteristics into filter, wrapper, embedded, hybrid, and ensemble categories [3], [4]. Table 1 provides an overview on recent gene selection methods according to this classification.

**Table 1:** Overview on traditional gene selection approaches and their classification. Filter approaches have lowest complexity, wrapper and embedded approaches apply machine learning strategies, hybrid and ensemble approaches combine multiple approaches.

Category	Functionality	Characteristics	Approaches
filter	uses only	+ independent of classifier	Information Gain (IG) [8]
	intrinsic data	+ low complexity	ReliefF [9]
	characteristics	+ good generalization	and [10], [11], [12], [13], [14], [15]
wrapper	uses learning	+ detects gene dependencies	Genetic Algorithms (GA) [16]
	algorithm to	/ interacts with classifier	Successive Feature
	evaluate genes	− high complexity	Selection (SFS) [17]
		− risk of overfitting	
embedded	gene selection	+ detects gene dependencies	Support Vector Machine
	is embedded into	/ interacts with classifier	with Recursive Feature
	learning algorithm		Elimination(SVM-RFE) [18] and [19], [20]
hybrid	applies multiple	/ intermediate complexity	SVM-RFE + mRMR Filter [21]
	approaches	− risk of slight overfitting	and [22], [23]
	sequentially		
ensemble	uses aggregate	+ good for small	Ensemble Gene Selection
	of a group of	sample domains	by Grouping (EGSG) [24]
	gene rankings	+ less prone to overfitting	and [25], [26]
		− computationally expensive	
		− difficult to interpret	

While the simplest statistical methods (filter) are in favor for feasibility and usability reasons, more complex approaches achieve a higher result set accuracy by applying machine learning methods (wrapper and embedded) or combining multiple gene selection methods (hybrid and ensemble).

A simple, yet common filter gene selection approach is based on the variance of all genes across samples. This variance-based (VB) approach assumes that highly variant genes are likely to separate samples into distinct groups. Genes are therefore ranked according to their variance in decreasing order.

As another example, Information Gain (IG) is defined as “the difference between the prior uncertainty and expected posterior uncertainty using” a certain feature [8]. The information gain of a gene is calculated using the change in entropy of a dataset given this gene is known. However, information gain is a univariate approach, i.e. it considers each gene independently. As biological processes consist of gene interactions, univariate filters like information gain cannot adequately reflect and identify the underlying biological processes in the data. ReliefF is a more sophisticated filter approach that can also deal with noisy and incomplete data and multi-class problems [9]. It builds on and extends the original, instance-based Relief feature selection approach, which works by randomly choosing one instance of a dataset and finding the nearest instance from the same and opposite class [27].

As wrapper and embedded gene selection approaches apply more complex machine learning strategies, they deliver more accurate results but tend to be viewed as a computational black box by its users. Support Vector Machines with Recursive Feature Elimination (SVM-RFE) is an embedded approach to create a feature ranking [18]. It repeatedly trains an SVM and removes the feature with the lowest weight magnitude. A feature subset ranking is produced when multiple features are removed at once. The usefulness of RFE compared to a naive feature ranking based on only the first iteration on RFE was demonstrated for small feature subsets over multiple classifiers.

Extensive studies from Bolón-Canedo et al. analyze the performance of multiple traditional gene selection methods on various datasets, both real-world and simulated [28], [29]. The authors highlight data characteristics challenging for traditional approaches, e.g. redundancy, noise, small sample to feature ratios, or class imbalances, and evaluate a wide range of gene selection approaches from all categories. Results of these studies show that subset filters, Information Gain, and SVM-RFE are advisable for scenarios with small sample to feature ratio like gene expression data. In addition, wrapper approaches have the highest computational costs, performed worst on average and are not applicable on small sample to feature ratio datasets due to overfitting.

### 2.2 Integrative Gene Selection

Integrative gene selection incorporates domain knowledge from external knowledge bases during its computations [4], [30]. Integrative gene selection leads to gene rankings that consider both the statistical significance of a gene in the dataset and the biological background information acquired through research.

Qi and Tang integrate Gene Ontology (GO) with a simple filter approach [5], [31], [32]. GO provides a unified, machine readable representation of genes and their products in three disjoint ontologies: Cellular Components, Molecular Functions, and Biological Processes. First, IG computes discriminative scores for each gene and discards genes with a score of zero. Second, all remaining genes are annotated with GO terms on biological processes. Third, GO terms receive a score that is the average of all the discriminative scores from the genes that are annotated with the respective term. From the highest ranked terms, the gene with the highest discriminative score is chosen for the final gene set, removed from the annotated genes, and the complete ranking and selection process for the GO terms and genes is repeated until the final gene set is complete. Compared to using IG only, Qi and Tang achieve better results on multiple cancer datasets.

SoFoCles integrates GO to find semantically similar genes [33]. A classic filter approach like *χ*^2^, ReliefF, or IG initially assigns a discriminative score to each gene. The top *n* ranked genes make up the initial set of candidate genes. All genes are annotated with GO terms and receive a similarity score. Genes with a high similarity score, i.e. that are semantically highly similar to candidate genes, are added to the set of candidates. Evaluation of SoFoCles shows that including biological knowledge in the gene selection process improves results.

Fang et al. combined the Kyoto Encyclopedia of Genes and Genomes (KEGG) and GO with IG [30]. KEGG is a pathway knowledge base providing manually curated pathways [6]. The authors apply IG as initial filter on the dataset and then search for GO and KEGG annotations for the remaining genes. To this annotation information, they then apply association mining and compute the interestingness of the frequent itemsets by averaging the original discriminative scores (from IG) of the contained genes. The final gene set is created by selecting the highest ranked gene from the top *n* frequent itemsets. They evaluated combinations of GO, KEGG, and both against IG and Qi and Tang’s approach. Although the new approach improved the overall accuracy only slightly, it was able to achieve this with a much lower number of genes.

Raghu et al. integrate KEGG, DisGeNET, and further genetic meta information into their approach. DisGeNET is a meta knowledge base on gene-disease-variant associations that integrates knowledge from curated databases [7]. Raghu et al. compute two metrics for a gene: the importance score and gene distance [34]. The importance score combines a gene-disease-association score from DisGeNET with the expression levels inherent in the data. The gene distance measures the distance between two genes according to their chromosomal locations and their associations to the same diseases. Both scores are then applied to find maximally relevant and diverse gene sets with Preferential Diversity (PrefDiv). In comparison to variance-based gene selection (VB), using the top *n* genes according to the formulated importance score performs similarly or slightly better in a predictive modeling task while selecting causally relevant genes.

Quanz et al. apply an integrative approach that is rather related to feature extraction, as they aim to map genes to pathways, which are then used as features for further pattern mining [6], [35]. They use the global test to extract pathways from KEGG related to the phenotypes of a dataset. The genes in each pathway are then transformed into one single feature by mean normalization or logistic regression. Experiments showed an improved performance over different traditional approaches, however the approach was not tested in a multiclass problem like cancer (sub-) type classification.

Acharya et al. replace genes as features by their GO terms [36]. They map all genes to GO terms and construct a gene-GO term matrix. On this matrix, Acharya et al. apply Partitioning Around Medoids (PAM) clustering. The best value *k* for the number of clusters is chosen by repeatedly calculating the silhouette index value of *k* clusters for multiple values of *k*. From those *k* clusters, the medoids are used as the selected gene subset. A comparison of the method to related approaches showed similar or better performance and the biological relevance of the chosen genes was proven.

Su et al. combine interaction networks with known breast cancer genes to identify potential drug targets [37]. Their Linear and Probabilistic Relations Prediction (LPRP) first creates a gene-gene interaction network from the given RNAseq data set and enhances it with network information from Pathway Commons [38]. The resulting interaction network is then filtered by known breast disease-related genes and their direct interaction partners to receive potential marker genes for breast cancer.

Most of the integrative approaches presented here have two things in common: They apply machine learning techniques, which again increases computational complexity, and they are highly specific to a single knowledge base, e.g. KEGG or GO. In contrast, we aim to flexibly combine low complexity traditional gene selection approaches with a multitude of knowledge bases.

## 3 Proposed Framework

To investigate the usefulness of the integration of biological knowledge in gene selection, we developed a framework for the flexible comparison of gene selection approaches that also incorporate external knowledge bases. The framework takes as input a gene expression dataset with some meta data, e.g. associated disease, and selected external knowledge bases. It outputs ranked lists of top *n* genes from selected gene selection approaches with their respective evaluation results, e.g. clustering accuracy.

According to Equation 1, we formally define the input of dataset as an expression matrix *E* of dimensions *m* by *n* where the set of samples *S* contains the sample IDs and the set of genes *G* contains the gene IDs.

(1) $$\begin{aligned}{S & =\{sample_{1},sample_{2},\ldots,sample_{m}\};|S|=m\cr G & =\{gene_{1},gene_{2},\ldots,gene_{n}\};|G|=n\cr E & =(expression_{sample_{i},gene_{j}})\in\mathbb{R}^{m\times n};\cr & \quad{}sample_{i}\in S;1\leq i\leq m;gene_{j}\in G;1\leq j\leq n}\end{aligned}$$

The elements of *E* contain the expression levels for the respective sample and gene. Additionally, *E* owns metadata that contains additional sample information such as disease code, tissue type, or clinical data. In this work, we include the disease assigned to a sample in our computations as we aim to classify samples by disease types. Equation 2 reflects this association with *D* as a set of possible diseases and *SDM* as a set of *m* mappings between *S* and *D*.

(2) $$\begin{aligned} D&=\{disease_{1},disease_{2},\ldots,disease_{o}\};\ |D|=o\\ SDM&=\{(sample_{1},disease_{x}),(sample_{2},disease_{y}),\ldots,(sample_{m},disease_{z})\};\\ &\quad{}sample_{i}\in S;\ 1\leq i\leq m;\ disease_{j}\in D;\ 1\leq j\leq o\end{aligned}$$

**Figure 1:**
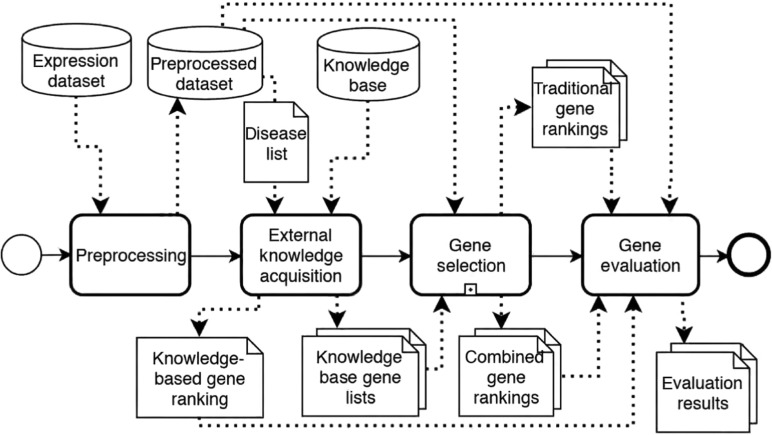
Overview on the analysis process carried out by our framework. It splits up into distinct steps of preprocessing, external knowledge acquisition, gene selection, and gene evaluation.

Our framework is divided into four distinct steps of a) data preprocessing, b) external knowledge acquisition, c) gene selection and d) gene evaluation. Figure 1 depicts the process of the framework in Business Process Model and Notation (BPMN) [39].

### 3.1 Preprocessing and External Knowledge Acquisition

We assume that the input dataset has been filtered, normalized by library size, and log-transformed prior. Our framework then checks the input data for the required format: Columns must contain gene names, rows must contain sample names. If this is not the case, the data is transformed accordingly. The framework then extracts disease codes from the metadata. This is straightforward if the expression metadata and all external knowledge bases used apply the same disease nomenclature. As this is seldomly the case, the framework creates separate disease ID lists for the respective knowledge bases, which are then used to query them. From each knowledge base, the framework then receives a list of relevant genes per disease. For each knowledge base, these gene lists are then combined into a separate list. We combine gene lists in an interleaving manner, this way preventing diseases with a long gene list or highly-scored genes to dominate the final gene list and representing each disease equally. Equation 3 depicts the formal definition of such a list *KBGL_i_*, with *gene_ix_* depicting gene *x* from knowledge base *i*.

(3) $$\begin{aligned}KB&=\{knowledebase_{1},knowledgebase_{2},...,knowledgebase_{r}\};\ |KB|=r\\ KBGL_{i}&=\{gene_{i}1,gene_{i}2,\ldots,gene_{i}l\};\ |KBGL_{i}|=l;\ i\in KB\end{aligned}$$

### 3.2 Gene Selection

**Figure 2:**
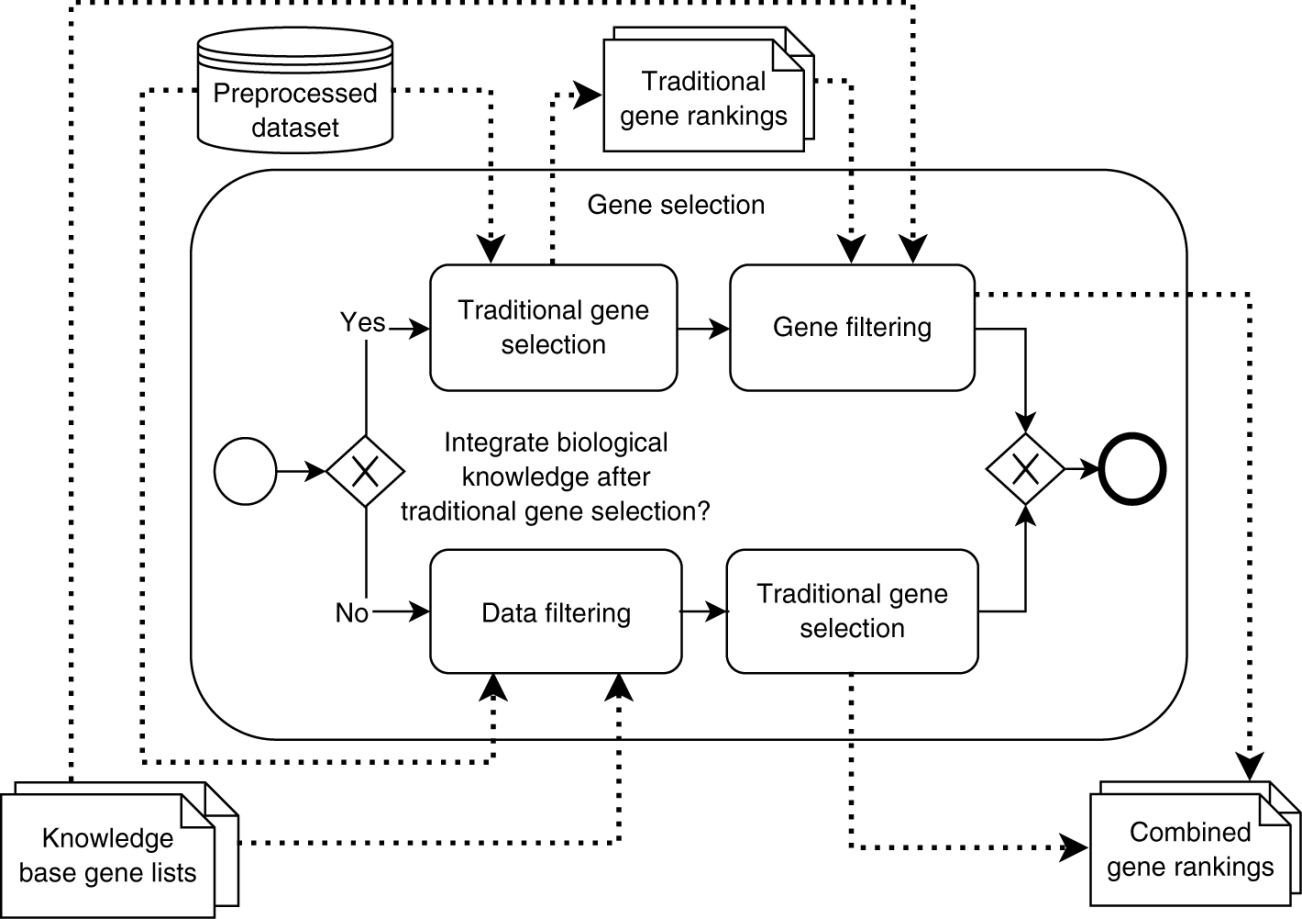
The actual gene selection step splits up into two alternatives data filtering and gene filtering, depending on when external knowledge is combined with traditional gene selection.

This step combines traditional gene selection approaches with external biological knowledge. It receives as input the preprocessed gene expression dataset *E* and the gene lists *KBGL_i_*, previously retrieved from external knowledge bases. It outputs gene rankings for the desired gene selection approaches and knowledge bases. Figure 2 proposes the two options how the external knowledge can be integrated into the gene selection process: Either in the form of *data filtering*, i.e. the external knowledge is integrated before the traditional gene selection takes place, or in the form of *gene filtering*, i.e. after the traditional gene selection.

#### 3.2.1 Data Filtering

Data filtering uses prior knowledge already before the actual gene selection takes place. Our framework filters the preprocessed dataset *E* in order to only retain the genes in *KBGL_i_*. Such a filtering can drastically reduce the dimensionality of the dataset and with that allow for more complex traditional gene selection approaches at low runtimes. Equation 4 shows how the framework restricts the set of genes *G* to *G_filtered_*: It intersects *G* with *KBGL_i_* to only keep the genes from the knowledge base *i*. The dataset *E* is then filtered by only retaining the genes from *G_filtered_*. The resulting *E_filtered_* is forwarded as input for the traditional gene selection.

(4) $$\begin{aligned}G_{filtered}&=G\cap KBGL_i\\ G_{filtered}&=\{fGene_{1},fGene_{2},\ldots,fGene_{p}\};\ |G_{filtered}|=p;p\leq l\\ E_{filtered}&=(expression_{sample_{i},gene_{j}})\in\mathbb{R}^{m\times p};\\ & \quad sample_{i}\in S;\ 1\leq i\leq m;\ gene_{j}\in G_{filtered};\ 1\leq j\leq p\end{aligned}$$

#### 3.2.2 Gene Filtering

**Figure 3:**
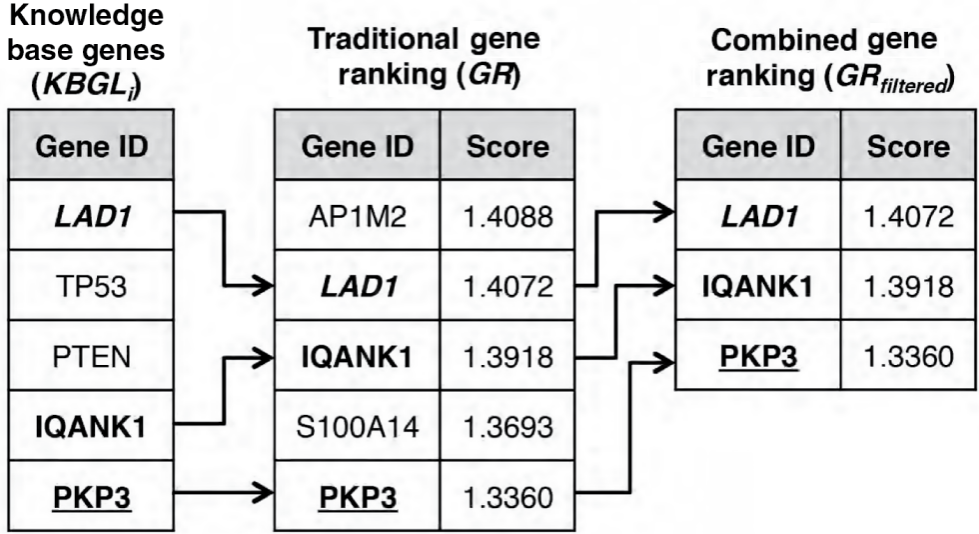
Gene filtering example showing the filtering of a traditional gene ranking by retaining only genes extracted from a knowledge base.

Gene Filtering employs external knowledge after the actual gene selection takes place. As shown in Figure 3, our approach filters gene selection produced by traditional gene rankings and keeps only those genes that are, according to prior research, connected to the diseases in the metadata. As defined in Equation 5, the traditional gene selection approach maps the expression dataset *E* to a gene ranking *GR*, which is an ordered set of discriminant scores for each gene in *G*. Our framework now aims to reduce *GR* to *GR_filtered_* by selecting only those genes that were returned by the external knowledge bases (*KBGL_i_*).

(5) $$\begin{aligned}& \quad\ \ f\colon E\to GR\\ GR& =(score_{gene_{x}},score_{gene_{y}},...score_{gene_{z}})\in\mathbb{R};gene_{i}\in G\\ GR_{filtered}& =(score_{gene_{l}},score_{gene_{m}},...score_{gene_{n}})\in\mathbb{R};gene_{i}\in G_{filtered}\end{aligned}$$

### 3.3 Gene Evaluation

This step evaluates the respective gene selection approaches according to standard evaluation metrics. It receives the gene expression dataset *E* as input and all gene rankings that were produced prior. Depending on the user’s choice, these can be gene rankings *GR* from traditional gene selection approaches or *GR_filtered_* from combinations with external knowledge bases. For each gene ranking, the framework selects the top *n* genes with 2 ≤ *n* ≤ 100 and uses them for training and classification on *E*. By choosing a wide range of different classifiers, e.g. SVM, Naive Bayes and Logistic Regression, users can make sure that the evaluated gene ranking achieves stable performance across different classifiers instead of suiting well for only one of them. Our framework conducts a 10-fold cross validation to reduce the effects of overfitting and reports a classification performance metric that is averaged over all classifiers.

### 3.4 Technical Implementation

**Figure 4:**
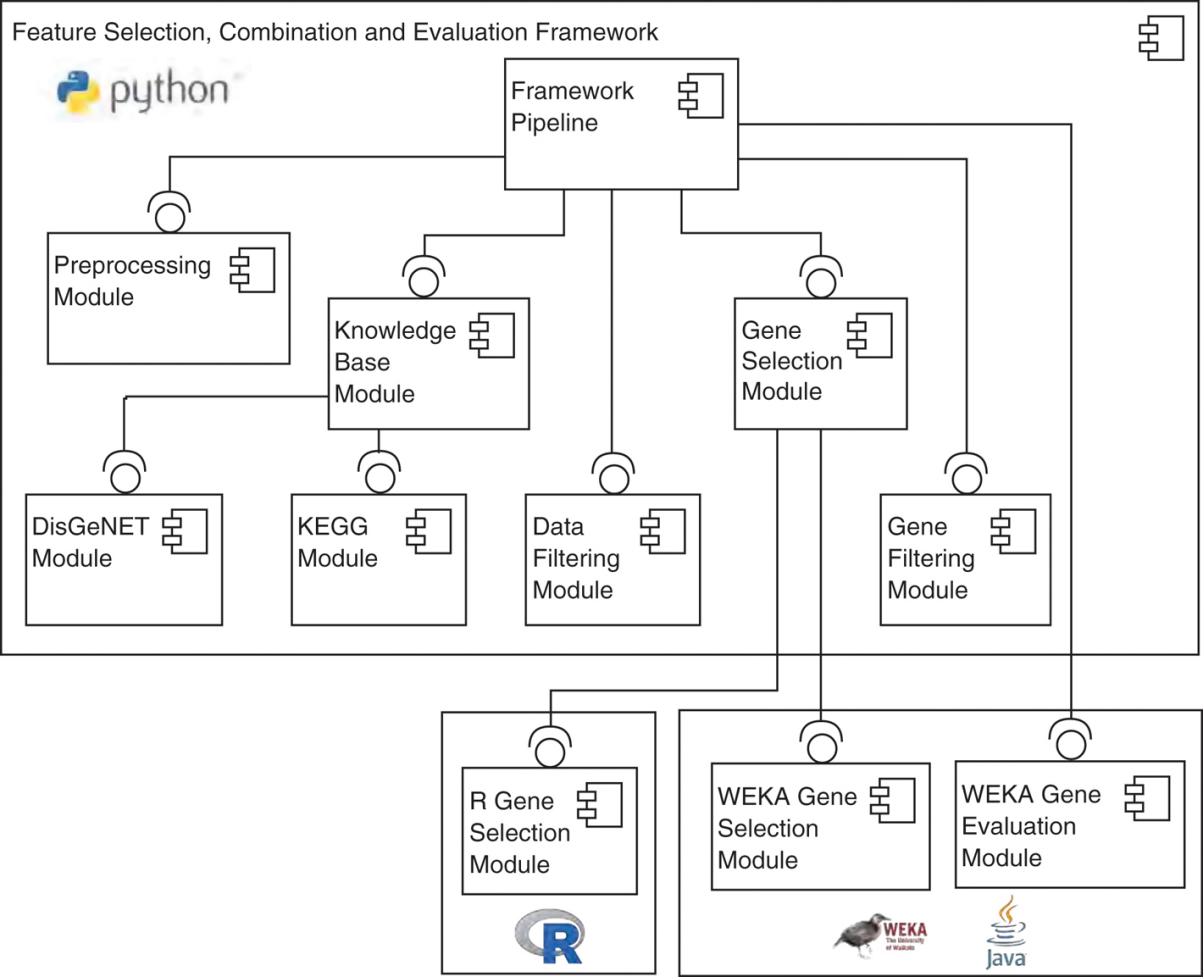
The architecture of the framework. Each processing step is encapsulated in an own module, with the Framework Pipeline module orchestrating the overall execution.

Our framework prototype is implemented in Python and automatically coordinates and executes the aforementioned processing steps. Figure 4 depicts the architecture of our prototype in Unified Modeling Language (UML) and shows respective modules for each of the processing steps [40]. Our framework currently provides modules for accessing DisGeNET and KEGG as external knowledge bases. It additionally owns modules for accessing WEKA and R, which provides users the flexibility to use any traditional gene selection, classifier and evaluation metric that is offered by WEKA or R [41]. If users want to integrate a new gene selection method that is not present in WEKA or R, they can either implement it as an own module within the framework or provide dedicated WEKA/R packages and extend the existing adapter module in our framework. To integrate a new knowledge base, users need to implement an own module that accesses the knowledge base’s respective Application Programming Interface (API). The framework is available on *https://github.com/CPerscheid/GSEF.git*. In the following, we briefly describe the main components of the system:

The Framework Pipeline is the main component of our framework and orchestrates the execution of all processing steps in their corresponding modules. It requires a configuration file with respective execution parameters, e.g. which knowledge bases and gene selection approach to use.

The Data Preprocessing Module prepares the raw dataset for subsequent processing. It currently integrates disease codes from metadata into the expression dataset to enable gene selection and evaluation. In the future, this module will be extended to include other standard preprocessing steps, e.g. filtering, normalization, or log-transformation.

The External Knowledge Base Module provides the interface for querying various external knowledge bases from within the framework. The corresponding submodules encapsulate the concrete API access of a specific knowledge base. Currently, submodules for KEGG and DisGeNET are implemented.

The Data Filtering Module creates a reduced expression dataset containing only genes present in the lists retrieved by the knowledge bases. Users can define whether they want to use a single specific knowledge base for data filtering or a combination of multiple knowledge bases.

The Gene Selection Module connects our Python framework with external implementations of traditional gene selection approaches. Currently, we have implemented modules for R and WEKA, respectively, but our framework can be easily extended for further resources by implementing a new submodule.

The Gene Filtering Module creates combined gene rankings from traditional gene selection approaches and the gene lists obtained from knowledge bases. It integrates external knowledge according to the procedures described prior.

The Gene Evaluation Module uses the previously produced gene rankings and evaluates their suitability for classification. This module uses the wide range of classifiers and evaluation metrics available in WEKA. For each gene ranking, this module measures the performance of using the top *n* genes with *n* increasing within a user-defined range. The final result of the framework is an evaluation for each distinct gene ranking containing a) the number of genes used, b) accuracies for each classifier, and c) averaged accuracy.

## 4 Experiments

We tested our framework and evaluated the proposed integration strategies against traditional gene selection methods. We selected gene selection approaches according to recommendations from Bolón-Canedo et al. [28], [29]. They conclude that IG, ReliefF and SVM-RFE are the best approaches in their respective categories of univariate filter, multivariate filter and embedded. We did not compare to wrapper approaches because of their high complexity and no clear advantages over filter or embedded methods. Additionally, we applied VB gene selection as the most simple, yet powerful approach for gene selection. We conducted the tests on a machine using four cores of an Intel^®^ Xeon^®^ CPU E5-2697 v3 running at 2.60 GHz and with 99 GB of main memory.

As external knowledge bases, we queried DisGeNET and KEGG and combined them with the aforementioned gene selection approaches. We constructed two datasets comprising expression values from 8 distinct cancer types each: Dataset I covers expression data from cancer types with good coverage in DisGeNET, dataset II cancer types with low coverage in DisGeNET. We assume a cancer type to have good coverage in DisGeNET if the top 5 Gene-Disease-Asssociations (GDAs) have a score greater than 0.2. We set this threshold because it guarantees that this association has been identified in at least one of the highest trusted resources, e.g. UniProt-KB/Swiss-Prot or CTD, or is covered by a sufficient mix of the other resources [7], [42], [43].

Table 2 provides details on the cancer types contained in datasets I and II, DisGeNET disease codes, and manually mapped KEGG pathway IDs applied when querying the knowledge bases. HNSC and SARC occur in different locations and tissue types and therefore no specific KEGG pathways exist for them. We downloaded data of The Cancer Genome Atlas (TCGA), filtered out all genes and samples that had more than 30 % missing values, normalized and log-transformed the data with variance stabilizing transformation (VST) [44], [45]. After preprocessing, Dataset I spans 3189 samples and 55,572 genes, dataset II spans 3807 samples and 55,621 genes after preprocessing.

**Table 2:** Details on the two evaluation datasets and their corresponding cancer types, DisGeNET disease codes, and KEGG pathway IDs.

**TCGA**	**DisGeNET**	**KEGG**
**Cancer Type**	**Disease Code**	**Pathway ID**
**Dataset I**		
SARC – Sarcoma	C1261473	−
HNSC – Head and Neck Squamous Cell	C1168401	−
GBM – Glioblastoma Multiforma	C0017636	hsa05214
KIRC – Kidney Renal Clear Cell Carcinoma	C0279702	hsa05211
LAML – Acute Myeloid Leukemia	C0023467	hsa05221
LUAD – Lung Adenocarcinoma	C0152013	hsa05223
THCA – Thyroid Carcinoma	C0238463	hsa05216
UCEC – Uterine Corpus Endometrial Carcinoma	C0476089	hsa05213
**Dataset II**		
OV – Ovarian Serous Cystadenocarcinoma	C0279663	−
PRAD – Prostate Adenocarcinoma	C0007112	hsa05215
COAD – Colon Adenocarcinoma	C0338106	hsa05210
LUSC – Lung Squamous Cell Carcinoma	C0149782	hsa05223
BRCA – Breast Invasive Carcinoma	C0853879	hsa05224
PAAD – Pancreatic Adenocarcinoma	C0281361	hsa05212
STAD – Stomach Adenocarcinoma	C0278701	hsa05226
CESC – Cervical Squamous Cell Carcinoma and Endocervical Adenocarcinoma	C0279671	hsa05165, hsa05203

We evaluated the selected approaches according to the following criteria: Classification accuracy, runtime performance (dataset I only), and overlap of gene rankings (dataset I only). Regarding classification accuracy, we used 10-fold cross validation with SVM, Logistic Regression, Naive Bayes and KNN (with *k* = 3; 5) and averaged accuracy across classifiers. For the comparison of execution time, we measured 10 runs of 10 executions for each gene selection method, computed the standard deviation and selected the run with lowest standard deviation as final measurement.

### 4.1 Classification Accuracy

We measured classification accuracy for both datasets according to the described setup. We compared traditional gene selection approaches with their combined versions, i.e. data filtering and gene filtering, applying DisGeNET and KEGG as knowledge bases.

#### 4.1.1 Results for Dataset I

**Figure 5:**
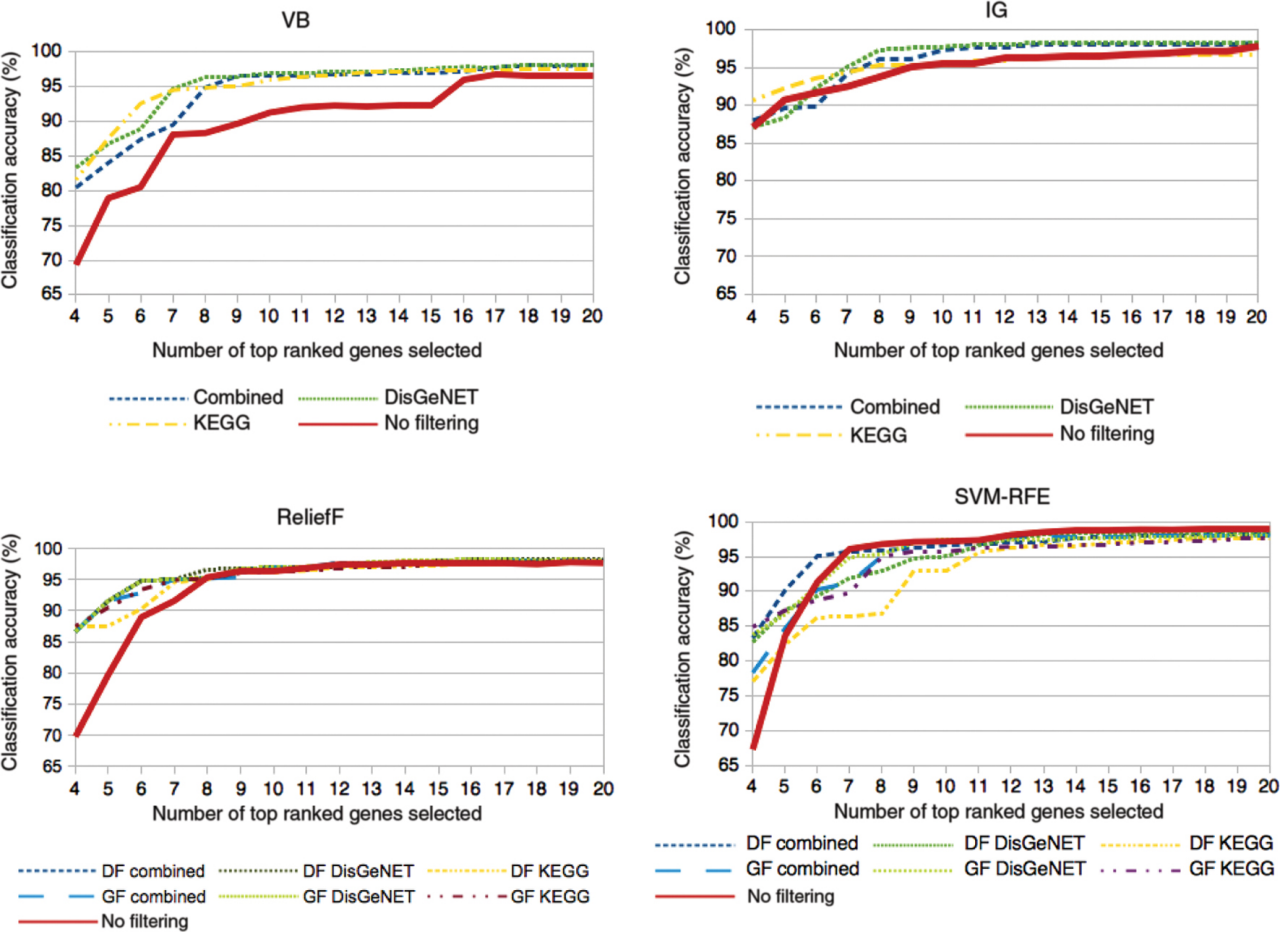
Classification accuracies on dataset I for the respective traditional gene selection approaches and their combination with external knowledge: Variance-based (VB), Information Gain (IG), ReliefF, and SVM-RFE.

Figure 5 shows classification accuracies for the four selected traditional gene selection approaches and their combinations with KEGG and DisGeNET. From the presented accuracy performances, we observe that all approaches reach a high classification accuracy already between 96 and 99 %. In general, combined approaches yield significantly higher classification accuracies already for the lower gene set sizes, starting with accuracies between 80 and 90 %. While the accuracies vary more widely between approaches for smaller numbers of genes selected, all approaches eventually converge to plateaus that are very close to each other, mostly around 7–8 genes.

As VB is a univariate filter that considers each gene independently from each other, data and gene filtering will produce the same gene rankings. Therefore, we only state the type of knowledge base, but not whether data or gene filtering were applied. According to the evaluation results, all approaches for VB achieve classification accuracies between 95 and 98 %. All combinations of VB with external knowledge bases improve classification accuracy significantly for gene sets of up to 16 genes. For larger gene sets, combined approaches still perform better than traditional VB, but with only a minor improvement. All combined approaches reach a classification accuracy above 95 % already for 9 genes, whereas the traditional VB approach reaches this accuracy with 16 genes. A combination with DisGeNET leads to best classification accuracies, although there are only minor accuracy differences between combined approaches.

For IG, all approaches reach high classification accuracies above 90 % already with a low number of genes and increase to accuracies up to 98 %. As IG is also a univariate filter as VB, the same applies regarding results for data and gene filtering. In contrast to VB, IG delivers better classification accuracies in general and catches up with a combination with KEGG for gene sets larger than 8 genes. A combination with DisGeNET outperforms all other approaches for gene sets larger than 7 genes. A combination of both knowledge bases, however, does not stabilize these results.

In contrast to VB and IG, ReliefF is a multivariate filter approach, i.e. genes are selected according to their relations to other genes. Consequently, applying data filtering (DF) and gene filtering (GF) will yield different results. Classification accuracies for the original ReliefF approach are outperformed by combined approaches throughout all gene set sizes. Gene filtering with DisGeNET performs best in general and reaches classification accuracies of up to 98 %. In general, all combined approaches show very similar performances once they reached a plateau.

Unlike the previous approaches, the original SVM-RFE outperforms its combined approaches for gene set sizes larger than 6. It reaches a classification accuracy of 95 % for already 7 genes, which rises up to 99 % classification accuracy. From the combined approaches, again a combination with KEGG performs worst.

**Figure 6:**
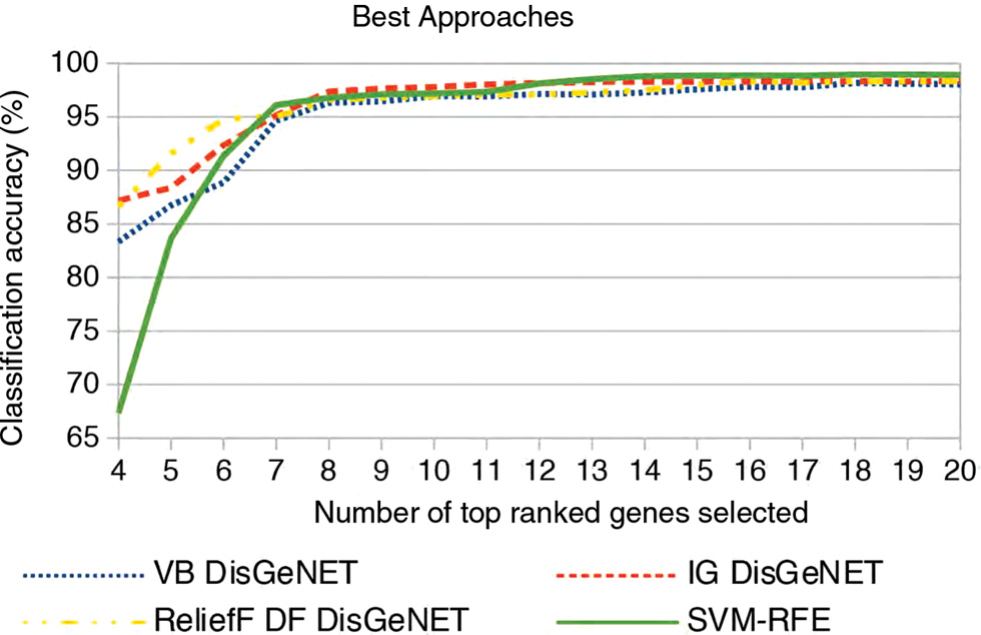
Classification accuracies on dataset I of the best performing approaches for the respective traditional gene selection approach.

Figure 6 compares the top performing approaches from our previous comparisons between traditional gene selection and their combinations with external knowledge bases. From each comparison, we choose the approach with the highest average classification accuracy. For VB, IG, and ReliefF, a combination with DisGeNET always achieves best results, whereas SVM-RFE is the only traditional gene selection approach that outperforms its combinations with external knowledge bases. Results show that integrative approaches based on simpler filter approaches outperform SVM-RFE up until 12 genes selected and achieve competable classification accuracy for larger gene sets.

#### 4.1.2 Results for Dataset II

**Figure 7:**
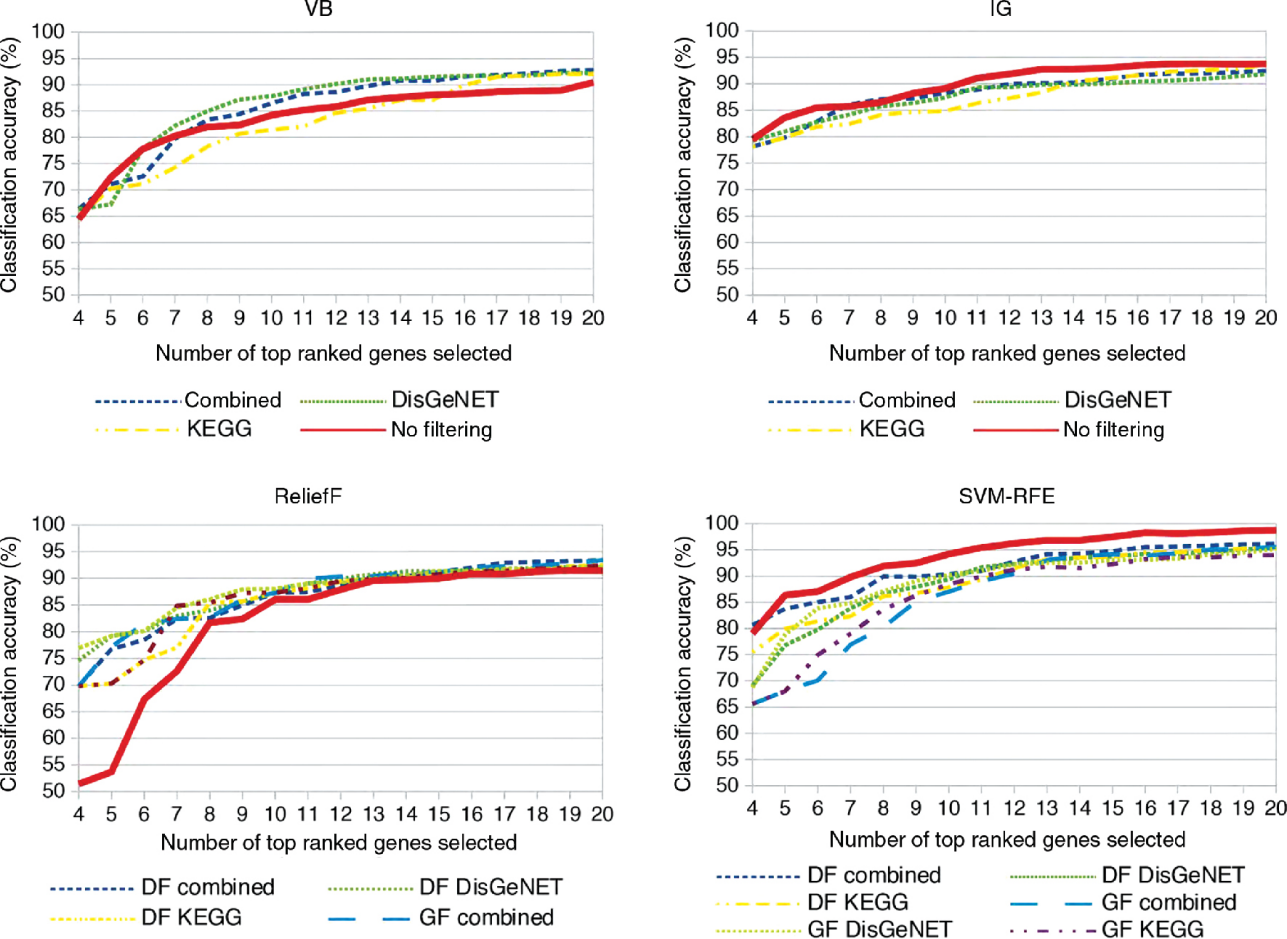
Classification accuracies on dataset II for the respective traditional gene selection approaches and their combination with external knowledge: Variance-based (VB), Information Gain (IG), ReliefF, and SVM-RFE.

With our evaluations on classification accuracies for dataset II, we aim to examine how our combined approaches perform for datasets on diseases that are not well researched. Figure 7 depicts classification accuracies for VB, IG, ReliefF, and SVM-RFE and compares them to their combinations with external knowledge bases, respectively. Similar to results for dataset I, we also observe a convergence of all approaches to a plateau. In contrast to dataset I, the overall best accuracy is lower with up to 90–95 %, the variance between approaches is larger, and the plateaus are reached later for larger gene sizes.

Although DisGeNET coverage is low for this dataset, VB is still outperformed by its combined approaches for almost all gene set sizes. Again, using DisGeNET seems to deliver a gene set more suitable for classification than KEGG, as the latter procedure scores lowest from the combined approaches.

Classification accuracies of all IG approaches increase up to 92–94 %. In contrast to VB, the original IG approach this time performs best for all gene set sizes except for 7 and 8 genes. For most of the gene set sizes, a combination with KEGG achieves lowest classification accuracies.

Similar to IG, ReliefF and its combined approaches reach a classification accuracy between 92 and 94 %. The original ReliefF approach, however, is always outperformed by combined approaches, which altogether achieve very similar classification accuracies.

For SVM-RFE, all approaches reach highest classification accuracies up between 94 and 98 %. In contrast to the other traditional approaches, the original SVM-RFE approach performs significantly better than all combined approaches for gene set sizes above 5. From the approaches integrating external knowledge, data filtering with a combination of both knowledge bases achieves best results, hence the original SVM-RFE performs significantly better.

**Figure 8:**
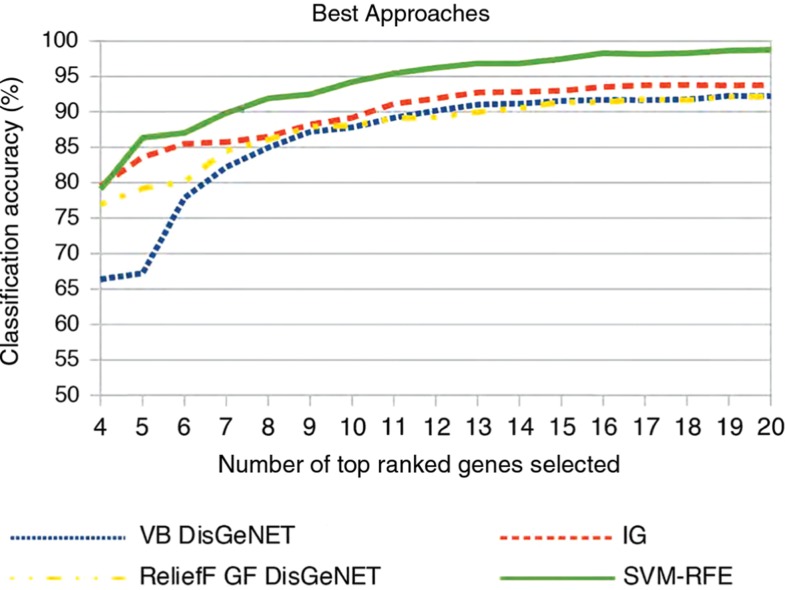
Classification accuracies on dataset II of the best performing approaches for the respective traditional gene selection approach.

Figure 8 compares the top performing approaches from our previous comparisons between traditional gene selection and their combinations with external knowledge bases. From each of the presented comparisons, we chose the approach with the highest average classification accuracy. Results show that a combination with knowledge bases like DisGeNET does not pay off for all traditional approaches anymore. Moreover, SVM-RFE not only clearly outperforms its own combinatory approaches, but also all other traditional approaches and their combinations with knowledge bases.

### 4.2 Runtime Performance

**Figure 9:**
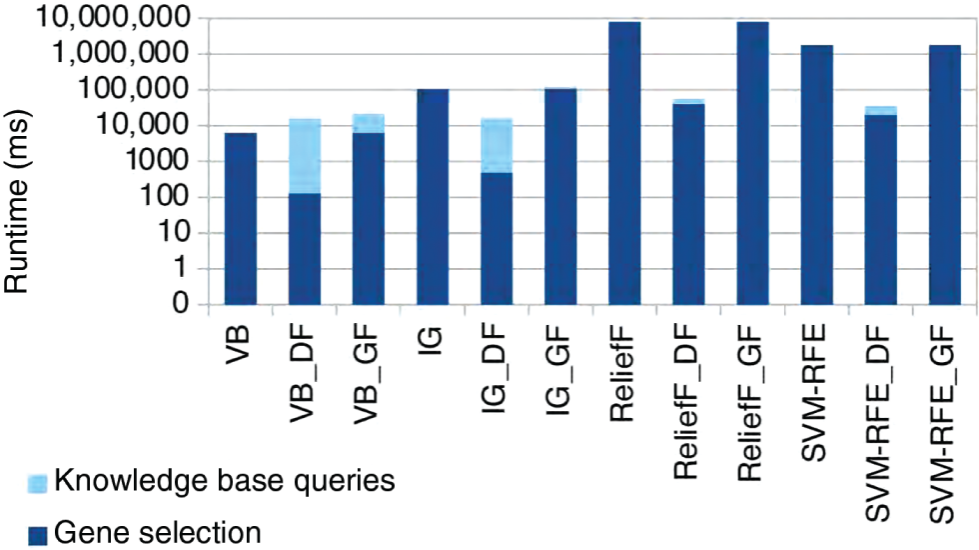
Runtime performances of traditional gene selection approaches compared to data and gene filtering with external knowledge bases.

Figure 9 compares execution runtimes on a logarithmic scale for traditional gene selection approaches and when data or gene filtering is applied. In general, the most complex gene selection approaches ReliefF and SVM-RFE have much higher execution runtimes than the simpler filter approaches. As expected, performance improvements for all approaches except VB are significant when applying data filtering. VB itself requires less runtime than querying an external knowledge base. In contrast, gene filtering increases computational runtimes, as external knowledge bases are queried additionally to the traditional gene selection approach. Still, execution runtimes for querying a knowledge base are only a fraction of the gene selection approaches’ runtimes and thus do not significantly increase the overall execution times. This impact can even be decreased by applying parallelization strategies, e.g. by querying external knowledge bases and carrying out gene selection in parallel instead of sequentially.

### 4.3 Gene Rankings Overlap

We applied multiple gene selection approaches on dataset I and examined the resulting gene rankings for any overlap of their top 100 genes. Our intuition was that there is an agreement to a certain extent by most of the gene selection approaches on specific genes that are discriminant for a particular disease. We assumed this portion to rise for integrative approaches that use the same external knowledge base.

**Figure 10:**
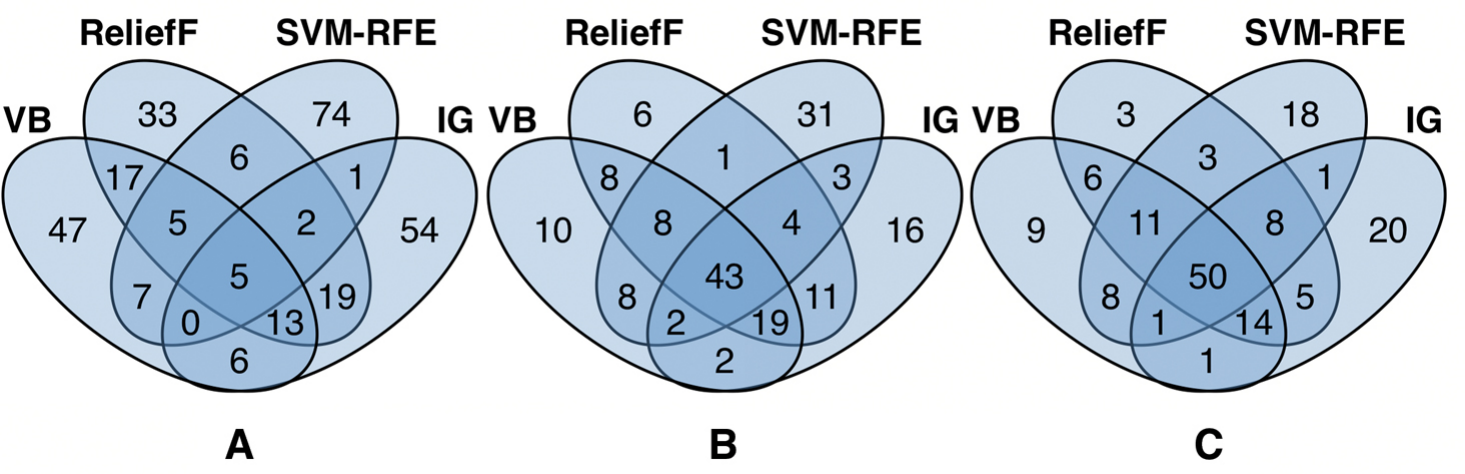
Overlaps of the top 100 genes selected by (A) traditional gene selection, (B) data filtering, and (C) gene filtering approaches.

Figure 10 depicts the overlaps of (A) traditional gene selection methods, (B) when applying data filtering, and (C) when applying gene filtering. From the traditional approaches, all of them jointly agree on only 5 genes. Moreover, filter methods, i.e. VB, ReliefF, and IG, have a higher overlap than SVM-RFE as embedded approach. SVM-RFE in general shows only a small overlap of 26 genes with other approaches. Agreement on the selected genes rises as expected when applying data filtering. Here, filter gene selection approaches have an increased total overlap between 84 and 94 genes whereas SVM-RFE agrees on only 69 genes. This effect even increases when applying gene filtering, where now all gene selection approaches agree on 50 out of 100 top ranked genes.

## 5 Discussion

Our experiment results show that integrating biological knowledge into the gene selection process improves classification accuracy, execution runtime and consistency of selected genes.

Especially simpler filter approaches, e.g. VB and IG, profit from the integration of external knowledge. They now provide classification accuracies that can compete with more complex approaches like SVM-RFE. This fact, together with far less computational runtimes and a computation procedure that remains transparent and interpretable for users, renders an integrative approach feasible for its application in practice also on larger datasets. SVM-RFE, on the other hand, does not require external knowledge to achieve best classification performances across datasets and gene selection approaches. Moreover, integrating external knowledge can even decrease its performance significantly. Together with the performance differences to filter approaches, this shows that more complex machine learning based gene selection approaches like SVM-RFE interpret the data fundamentally different than filter methods. Although these methods have proven to work out on real-world data, the appeareance as a black box together with increased computational runtimes renders it unattractive for potential users.

Our investigations regarding overlaps on selected genes also revealed interesting results. The traditional gene selection approaches have a low overlap in general. The slightly higher overlap between filter results again indicates their similarities in computing gene rankings compared to more complex approaches like SVM-RFE. The generally high classification accuracies of all gene selection approaches suggests that they all are likely to detect most of the underlying biological processes, but select different or multiple representatives. The generally low agreement on selected genes has been recognized and discussed in former studies [46], [47], [48], [49]. This instability of gene selection approaches across methods and datasets puts the validity of their produced gene rankings in question. Literature assumes that the integration of external knowledge can stabilize gene selection and lead to more consistent results [50], [51]. The increased overlap when combining external knowledge bases suggests that this can indeed be the case.

We evaluated all approaches with two datasets representing diseases that have good and bad coverage in knowledge bases, respectively. Integrating external knowledge proved to increase accuracies for the dataset with well covered diseases. For less covered diseases, combined approaches showed both a slight improvement and decline in classification accuracies, depending on the traditional approach. Real-world datasets will most likely contain a mix of well and less researched diseases. Based on our evaluation results, we assume that if our integrative is applied in practice, it will still have a positive impact on classification accuracy.

From the knowledge bases we integrated, DisGeNET appeared to yield best classification improvements. Integrating KEGG, on the other hand, even showed worse results than the traditional approach. One reason for this behavior could be our selection of the diseases contained in the datasets based on their coverage in DisGeNET alone. A different composition of diseases could have led to more favorable results for KEGG. On the other hand, we have to bear in mind that DisGeNET is a meta knowledge base providing gene-disease associations, i.e. integrates information from various curated knowledge bases on direct associations of a gene to a disease. In contrast, KEGG provides a far smaller set of self-curated pathways, i.e. interaction data between genes, gene products, or other complexes. This difference might render DisGeNET more suitable for a straightforward integrative approach as we presented here. However, we assume that an integration of KEGG can achieve better classification accuracy if gene interactions are more thoroughly integrated into the gene selection process.

### 5.1 Threats to Validity

Our evaluation of the presented approach comprises only a subset of possible application scenarios, which limits the validity of our findings to this specific setting. A more in-depth study could show even better results for combinations or reveal cases where the integration of biological knowledge is especially useful.

We tested our approaches with two cancer datasets that have either good or bad coverage in knowledge bases. Further experiments should include datasets containing diseases other than cancer and that have both good and bad coverage on the knowledge bases. In addition, we selected the data based on its coverage in DisGeNET alone, although also integrating KEGG. This might be one reason why combinations with DisGeNET almost always performed best. We also only integrated two knowledge bases, although there are many others with high reputation, e.g. GO. A combination with other knowledge bases is thus likely to perform different and should be tested. Finally, we only compared a fraction of the existing traditional gene selection approaches. Future experiments should also include wrapper, ensemble, or hybrid approaches to receive a comprehensive view.

Our framework enables and encourages an extension of the experiment settings as it can be easily enhanced by further gene selection methods and external knowledge sources. We think, however, that the results obtained with our current experiments already point towards the right direction.

### 5.2 Limitations

There are some limitations to the proposed integrative approaches though. Our approach works in a supervised fashion: We always need some kind of labels in the data to use them for querying external knowledge bases. In addition, the labels must be suitable query parameters for the knowledge bases. For example, DisGeNET only delivers results if the query term is a disease, gene, or variant.

For receiving reasonable results, integrative approaches like the ones presented here require knowledge bases with an extensive coverage of the questionable domain. As shown in our evaluations, our approaches perform worse for diseases that are not well researched and thus not sufficiently covered by the knowledge bases. This makes an integrative approach as presented here not applicable for analyzing data on rare diseases. However, as biological knowledge bases gather more data through research, integrative approaches in general will become more viable and useful for an increasing number of scenarios in the future.

Integrative approaches that automatically include external knowledge bases depend on the accessibility of the respective knowledge bases. Most knowledge bases offer RESTful APIs for online access, which avoids downloading data locally, but also requires an internet connection and maintenance of the whole approach once the API gets updated.

Lastly, our presented integrative approach leads to a substantial filtering of the feature space. The experiments in this work show the benefits regarding runtime, classification performance and consistency of selection. However, this straightforward combination of gene expression with external data runs into danger of rather representing the knowledge base and not the actual data, and it also impedes the detection of unassociated genes and their correlations. Future approaches should thus integrate external knowledge in a way that carefully balances both latest research findings and insights from statistical data analysis.

## 6 Conclusion

In this work, we examined the usefulness of utilizing information from biological knowledge bases in the gene selection process for gene expression data. We tested our hypothesis with a straightforward integration of external knowledge into gene selection and presented a corresponding framework for comparing both traditional and knowledge based gene selection methods for gene expression data.

Our results show that especially simple traditional filter approaches, e.g. IG, profit from the integration of external data and can now compete with more complex machine learning approaches at nearly equal classification accuracy, but far less computational runtime and a more transparent and thus interpretable computation process.

Future work will include an extension of the framework and experiment setting to include further external knowledge bases, both traditional and integrative gene selection approaches, and more datasets.
